# Species-specific pharmacology of maximakinin, an amphibian homologue of bradykinin: putative prodrug activity at the human B_2_ receptor and peptidase resistance in rats

**DOI:** 10.7717/peerj.2911

**Published:** 2017-01-18

**Authors:** Xavier Charest-Morin, Hélène Bachelard, Melissa Jean, Francois Marceau

**Affiliations:** 1 Axe Microbiologie-Infectiologie et Immunologie, CHU de Québec-Université Laval and Université Laval, Québec, QC, Canada; 2 Axe endocrinologie et néphrologie, CHU de Québec-Université Laval and Université Laval, Québec, QC, Canada

**Keywords:** Bradykinin B2 receptor, Maximakinin, MRGPRX2, Human umbilical vein, Angiotensin converting enzyme, *In vivo* hemodynamics

## Abstract

Maximakinin (MK), an amphibian peptide possessing the C-terminal sequence of bradykinin (BK), is a BK B_2_ receptor (B_2_R) agonist eliciting prolonged signaling. We reinvestigated this 19-mer for species-specific pharmacologic profile, *in vivo* confirmation of resistance to inactivation by angiotensin converting enzyme (ACE), value as a module for the design of fusion proteins that bind to the B_2_R in mammalian species and potential activity as a histamine releaser. Competition of the binding of [^3^H]BK to recombinant human myc-B_2_Rs in cells that express these receptors revealed that MK possessed a tenuous fraction (<0.1%) of the affinity of BK, despite being only ∼20-fold less potent than BK in a contractility assay based on the human isolated umbilical vein. These findings are reconciled by the generation of C-terminal fragments, like Lys-Gly-Pro-BK and Gly-Pro-BK, when the latent MK is incubated with human venous tissue (LC-MS), supporting activation *via* hydrolysis upstream of the BK sequence. At the rat recombinant myc-B_2_R, MK had a lesser affinity than that of BK, but with a narrower margin (6.2-fold, radioligand binding competition). Accordingly, MK (10 nM) stimulated calcium transients in cells that expressed the rat receptors, but not the human B_2_R. Recombinant MRGPRX2, a receptor that mediates cationic peptide-induced mast cell secretion, minimally responded by increased [Ca^+2^]_i_ to MK at 10 µM. Enhanced green fluorescent protein fused to MK (EGFP-MK) labeled cells that expressed rat, but not human B_2_Rs. Intravenous MK induced dose-dependent hypotensive, vasodilator and tachycardic responses in anesthetized rats and the effects were antagonized by pretreatment with icatibant but not modified by pyrilamine or enalaprilat. Strong species-specific responses to the toxin-derived peptide MK and its prodrug status in the isolated human vein were evidenced. Accordingly, MK in the EGFP-MK fusion protein is a pharmacophore module that confers affinity for the rat B_2_R, but not for the human form of the B_2_R. MK is unlikely to be an efficient mast cell activator, but its resistance to inactivation by ACE was confirmed *in vivo*.

## Introduction

We recently reported that maximakinin (MK, also called bombinakinin M), an amphibian 19-mer peptide possessing the C-terminal sequence of bradykinin (BK), is an atypical BK B_2_ receptor (B_2_R) agonist (Bawolak et al., 2012). MK exerts prolonged B_2_R-mediated signaling (≥12 h; ERK1/2 phosphorylation, c-Fos induction, receptor endocytosis and downregulation) in a cellular system maintained in serum-containing medium possessing ACE and other peptidase activities, whereas the brief effects of BK were no longer detectable 12 h post-stimulation (Bawolak et al., 2012). Further, MK has been proposed as a module for the design of fusion proteins that bind to this receptor type in mammalian species, such as enhanced green fluorescent protein-MK (EGFP-MK) (Charest-Morin et al., 2013). Angiotensin converting enzyme (ACE) is the major peptidase that disposes of BK in the extracellular space and initially removes a C-terminal dipeptide from BK (Cyr et al., 2001; Fryer et al., 2008). MK has substantially less affinity for ACE than BK ([^3^H]enalaprilat displacement from recombinant ACE; Bawolak et al., 2012) and EGFP-MK, none, although BK, MK and EGFP-MK share the same C-terminal sequence. N-terminal prolongation of BK has been shown to decrease inactivation by ACE in other instances (Roblero, Ryan & Stewart, 1973), possibly due to exclusion of large peptides/proteins by this carboxydipeptidase.

MK, encoded by two genes as five identical and cleavable amino acid sequences (Chen et al., 2003), is presumably a dissuasive toxin expressed in the skin of the toad *Bombina maxima*. Several such BK-related peptides of amphibian origin are believed to have co-evolved with the kinin receptors of the predatory species such as birds and snakes (Xi et al., 2015). Further, *Polistes* kinin, from a wasp venom, is a comparable peptide possessing the BK sequence at its C-terminus and possessing a hydrophilic N-terminal extension; it has been reported a long time ago that *Polistes* kinin releases histamine from mast cells, but that BK doesn’t (Johnson & Erdös, 1973). In another study, BK released histamine from rat mast cells with an EC_50_ of 17 µM, and Lys-BK of 7.7 µM (Devillier et al., 1985). BK possesses two amino acid residues with a basic side chain, whereas Lys-BK possesses three, MK four and *Polistes* kinin five such residues. The mode of action of many cationic peptides that are mast cell releasers has been recently elucidated: they activate a common G protein coupled receptor termed MRGPRX2 that is expressed in the mature mast cell of connective tissue (McNeil et al., 2015).

We have reexamined the pharmacology of MK with several objectives: (1) The species-dependent variation of the pharmacology of MK, intact or included in the sequence of a fusion protein, has been assessed in two mammalian species, rats and humans; (2) The pro-drug status of the N-terminally extended BK sequences MK has been examined because it may possess a low affinity for B_2_Rs from specific mammalian species and release shorter and more active peptides following cleavage upstream of the BK sequence. This approach is inspired from our recent work on C-terminally extended sequences of BK that act as prodrugs and are activated by carboxypeptidases in vascular tissue and *in vivo* (Charest-Morin et al., 2014; Jean et al., 2016a); (3) While intravenously injected BK is massively inactivated by ACE in rats (Jean et al., 2016a), we verified the prediction that pharmacologic ACE inhibition does not influence hemodynamic responses to MK owing to its natural resistance to this major kininase; (4) A direct action on the mast cell receptor MRGPRX2 would add to the toxicity of MK in a manner independent from kinin receptors; this has been investigated using the human recombinant form of this mast cell receptor and an antihistamine drug in other assays. Altogether, the objectives supported the evaluation of MK as a possible B_2_R-binding module for the design of diagnostic or therapeutic fusion proteins that would be applicable to both preclinical models of cardiovascular disease (thus, inclusion of the rat) and human medicine with no off-target side effects (thus, inclusion of the mast cell receptor).

## Materials and Methods

### Drugs

BK was purchased from Bachem (Torrance, CA, USA), the B_2_R antagonist icatibant, from Phoenix Pharmaceuticals (Burlingame, CA, USA), enalaprilat dehydrate, from Kemprotec Ltd. (Maltby, Middlesbrough, UK) and MK from Tocris Bioscience (Minneapolis, MN, USA; sequence of BK-related peptides in Table 1). MK-des-Arg (MK sequence without the C-terminal Arg residue) has been synthesized by Peptide 2.0 Inc. (Chantilly, VA, USA) *via* standard solid-phase methodology and provided as a >99% pure reagent (mass spectroscopy and HPLC analyses). The activity of MK-des-Arg has recently reported at the human BK B_1_ receptor (Charest-Morin & Marceau, 2016). The other drugs were purchased from Sigma-Aldrich (St. Louis, MO, USA). Compound 48/80 is the condensation product of N-methyl-p-methoxyphenethylamine with formaldehyde and is a mixture of 3- to 6-mers as supplied (Sigma-Aldrich).

**Table 1:** Primary structure of bradykinin (BK)-related peptides exploited in the present work.

Agonist peptide	Sequence	Molecular weight	[^3^H]BK binding competition assay: IC_50_ values (nM) [95% C.L.]		Human umbilical vein EC_50_ (nM) [95% C.L.]
			Human B_2_R	Rat B_2_R	
BK	RPPGFSPFR	1059.6	5.16 [4.1–6.5]	19.2 [12.5–29.5]	6.9 [3.8–12.2]
Maximakinin (MK)	DLPKINRKGPRPPGFSPFR	2179.4	7,560 [5,060–11,300]	120 [92–155]	145 [106–197]
MK-des-Arg	DLPKINRKGPRPPGFSPF	2024.2	>10,000	–	>10,000

### Receptor constructions, radioligand binding assays

The construction of myc-tagged B_2_R expression vectors containing the human and rat receptor sequence has been recently reported, as well as the derivation of HEK 293a cell lines that stably express each one of these constructions (Charest-Morin et al., 2015). The HEK 293a cell line was originally obtained from Sigma-Aldrich. Affinity of N-terminally extended kinins for the B_2_R was evaluated using a radioligand binding competition assay performed at 0 °C in the presence of peptidase inhibitors that included captopril and PMSF (Houle et al., 2000). Briefly, the binding of 3 nM [^3^H]bradykinin (Perkin Elmer Life Sciences; 90 Ci/mmol) to adherent intact HEK 293a cells stably expressing a myc-B_2_R construction was applied to construct binding competition curves for a series of unlabeled  peptides.

The vector MRGPRX2-Tango (human receptor sequence; Kroeze et al., 2015) was a gift from Dr. Bryan Roth (Addgene plasmid # 66440). This vector was transiently expressed in HEK 293a cells using the MegaTran transfection reagent as directed by the supplier (Origene). The encoded receptor notably signals *via* calcium and responds to the mast cell activator, compound 48/80 (Kroeze et al., 2015; McNeil et al., 2015).

### Calcium mobilization

To quantify the calcium mobilization induced by kinins, Fura-2 fluorometry (Molecular Probes, Invitrogen Detection Technologies) was applied to HEK 293a cells stably expressing myc-B_2_R constructions possessing the human or rat sequence or to HEK 293a cell transiently transfected with the MRGPRX2-coding vector. Cells were cultivated in 75 cm^2^ flask and each flask allowed for three determinations. The cells were detached using enzyme-free Cell Dissociation Buffer (Life Technologies) resuspended in serum-free DMEM before being centrifuged 5 min at 1,100 rpm at room temperature. Following centrifugation, cells were resuspended in Hank’s balanced salt solution (1×, pH 7.4, prepared from 10× concentrate, Multicell Wisent, St. Bruno, Canada) with 10 mM HEPES and 1.6 mM CaCl_2_. At this point, Fura-2-AM was added to cell suspensions (final concentration 2 µM) and incubated 30 min in a 37 °C bath with agitation. After the incubation, cells were centrifuged and resuspended in the appropriate volume of buffer. Calcium mobilization was read with a thermostated (37 °C) spectrofluorimeter (SLM8000C; excitation 340 nm and emission 510 nm) in 2 ml suspension of cells loaded with Fura-2. After the readings, the maximum mean fluorescence (F_max_) was measured by adding 15 µL of 10% Triton X100 and the minimum mean fluorescence (F_min_) with further addition of 15 µl of NaOH 1 N and 100 µl of EGTA 100 mM. Calcium mobilization concentrations were established with the following equation ([Ca^+2^] = 224((y − F_min_)∕(F_max_ − y)), where y represents the fluorescence reading from the sample. To allow a better comparison of the results, the calcium mobilizations were expressed as intracellular Ca^2+^ fold increase *vs.* the baseline over time.

### Human umbilical vein contractility assay

The institutional research ethics board (CHU de Québec) approved the anonymous use of human umbilical cord segments obtained after elective cesarean section deliveries (file number: 2012-323). Informed written consent was obtained from mothers. Umbilical vein rings, used as a contractile bioassay for the BK B_2_R and histamine H_1_ receptor (H_1_R), were prepared and suspended in organ baths and submitted to equilibration in Krebs’ solution as described (Charest-Morin et al., 2014). The vascular preparation was used to assess the potency of individual peptides, the effect of B_2_R and H_1_R antagonists and of peptidase inhibitors (introduced 30 min before the agonist) on the apparent potency of MK and the kinetics of kinin-induced contraction, as outlined in Results.

### Liquid chromatography–mass spectrometry (LC–MS)

Synthetic MK (1 µM) was submitted to digestion in 2 ml of sterile-filtered Krebs solution in the presence of a freshly prepared ring of human umbilical vein (wet weight 25–37 mg). The tubes containing these materials were incubated at 37 °C for 5 or 15 min; the tissues were then removed and five volumes of cold ethanol (10 ml) were added to each tube. The tubes were incubated for 1 h on ice, centrifuged at 10,000 g to remove precipitated proteins and the kinin-containing supernatants were collected (procedure previously validated, Raymond et al., 1995). The supernatants were then completely evaporated to dryness in a Speedvac system and reconstituted in a small volume of water. Peptides were desalted on stage tip (C18), vacuum dried and then resuspended into 0.1% formic acid. One hundred fmol of each sample (starting MK quantity) was analyzed by mass spectrometry.

Mass spectrometry: peptide samples were separated by online reversed-phase (RP) nanoscale capillary liquid chromatography (nanoLC) and analyzed by electrospray mass spectrometry (ES MS/MS). The experiments were performed with a Ekspert NanoLC425 (Eksigent) coupled to a 5600+ mass spectrometer (AB Sciex, Framingham, MA, USA) equipped with a nanoelectrospray ion source. Peptide separation took place on a picofrit column (Reprosil 3u, 120A C18, 15 cm × 0.075 mm internal diameter). Peptides were eluted with a linear gradient from 5–35% solvent B (acetonitrile, 0.1% formic acid) in 35 min, at 300 nL/min. Mass spectra were acquired using a data dependent acquisition mode using Analyst software version 1.7. Each full scan mass spectrum (400 to 1,250 m/z) was followed by collision-induced dissociation of the twenty most intense ions. Dynamic exclusion was set for a period of 3 s and a tolerance of 100 ppm

Database searching: MGF peak list files were created using Protein Pilot version 5.0 software (Sciex) utilizing the Paragon and Progroup algorithms. MGF sample files were then analyzed using Mascot (Matrix Science, London, UK; version 2.5.1).

Mascot was set up to search the MK sequence assuming that the digestion enzyme was non-specific. Mascot was searched with a fragment ion mass tolerance of 0.100 Da and a parent ion tolerance of 0.100 Da. Deamidation of asparagine and glutamine and oxidation of methionine were specified in Mascot as variable modifications. Scaffold (version Scaffold_4.7.1; Proteome Software Inc., Portland, OR, USA) was used to validate MS/MS based peptide and protein identifications.

Data processing: Peak View 1.2.0.1 (Sciex) was used to extract the specific precursor masses and calculate the area under the curve. Extracted ion chromatoram (XIC) were generated from the TOF MS using a peak width of 0.05.

### Surgical preparation

All surgical and experimental procedures were reviewed and approved by the Animal Care and Handling Committee of Laval University, in accordance with the Canadian Council on Animal Care (file no. 2012-223). Experiments were performed on male Sprague-Dawley rats (300–375 g) purchased from Charles River Laboratories (St-Constant, Qc, Canada). Only animals of the male gender have been studied to be consistent with related and recently published data (Jean et al., 2016a). The rats were housed in a temperature-controlled room (22 ± 1 °C) on a 12 h/12 h light-dark cycle (lights on at 0600) and had free access to normal chow diet and tap water. They were allowed to acclimate to their environmental conditions for one week prior to being studied. At the end of the acclimation period, the rats were anesthetised with sodium pentobarbital (50 mg kg^−1^, i.p., supplemented as required) and had one catheter implanted into the right jugular vein (for intravenous ( i.v.) injection) and one into the left femoral artery (for direct and continuous measurement of blood pressure and heart rate as previously described, Jean et al., 2016a). In some experiments, a miniaturized pulsed Doppler flow probe (Haywood et al., 1981) was implanted around the distal abdominal aorta (below the level of the ileocaecal artery) through a midline abdominal incision to monitor changes in hindquarter hemodynamic, according to the method previously developed by Gardiner & Bennett (1988) and as previously described (Bachelard et al., 1992; Jean et al., 2016a). Experiments started at least 30 min following the end of surgery in anesthetised rats.

### *In vivo* hemodynamics in anesthetized rats

Baseline heart rate and phasic and mean arterial blood pressure were recorded over a period of 15 min in anesthetized rats. A dose response curve was then obtained by recording changes in blood pressure and heart rate elicited by i.v. injection of peptide vehicle followed by increasing doses of MK, or BK. Peptides were dissolved in isotonic saline (0.9% NaCl) containing 0.1% BSA to prevent the adsorption of peptide to the glassware and plastic surfaces. All i.v. injections were given as 100 µl boluses which were washed in with a further 100 µl of saline (the dead space of the catheter). Only one peptide was tested per group of rats and each injection started with saline-BSA 0.1% followed by the lowest dose of peptide. The next dose was administered once all recorded cardiovascular parameters had returned to baseline after the previous injection (usually 2–10 min). At the end of the experiments each animal was euthanized with an overdose of sodium pentobarbital (240 mg/kg, i.v.).

The mechanism subserving the cardiovascular responses to i.v. injections of increasing doses of MK was first investigated in animals pretreated with the ACE inhibitor, enalaprilat. In these experiments, enalaprilat was intravenously administered as bolus (0.1 mg/kg, 0.1 ml) following a 10 min period of baseline measurements of heart rate and blood pressure. Fifteen minutes later, a dose–response curve to MK was obtained, as described above. Further experiments were made in rats pretreated with a potent, long acting and selective B_2_R antagonist, icatibant (Hoe 140) (D-Arg-[Hyp^3^, Thi^5^, D-Tic^7^, Oic^8^] bradykinin) (Hock et al., 1991; Wirth et al., 1991; Rhaleb et al., 1992; Marceau et al., 1994). In these experiments, icatibant was intravenously administered as bolus (25 µg/kg, 0.1 ml) 15 min before the i.v. injection of increasing doses of MK, as above. Further dose–response curves were also obtained from rats pretreated with pyrilamine, a histamine H_1_R antagonist. In these experiments, pyrilamine was intravenously administered as bolus (5 mg/kg, 0.1 ml) 15 min before the i.v. injection of increasing doses of MK, as above. Control experiments were also performed to ensure that changes in blood pressure responses elicited by a high dose of histamine (6.4 µg/kg i.v.) could be inhibited by 5 mg/kg pyrilamine.

### Acute hindquarter hemodynamic effects in anesthetized rats

Before each experiment, a 30-min stabilization period was allowed, during which continuous recording of heart rate, phasic and mean blood pressure and phasic and mean Doppler shift signals from the hindquarter probe were made in anesthetized rats, using the Biopac data acquisition and analysis system previously described (Jean et al., 2016a). Then, a first group of rats (*n* = 8) received successive i.v. injections of peptide vehicle (saline-BSA 0.1%), BK (6.4 µg/kg), and MK (1.6 µg/kg). All i.v. injections were given as 100 µl boluses which were washed in with a further 100 µl of saline. The next dose was administered once all recorded cardiovascular parameters had returned to baseline after the previous injection (usually 5–7 min). Further experiments were made in a second group of rats (*n* = 6) pretreated with the B_2_R antagonist, icatibant. In these experiments, the antagonist was intravenously administered as bolus (25 µg/kg, 0.1 ml) followed 15-min later by the i.v. injections of peptide vehicle, BK (6.4 µg/kg) and MK (1.6 µg/kg), as described above. At the end of the experiments the rats were euthanized with an overdose of anesthetic (pentobarbital 240 mg/kg, i.v.).

### Microscopy

HEK 293a cells stably expressing myc-B_2_R constructions, either with human or rat sequence, were used for a side-by-side comparison of labeling by the EGFP-MK fusion protein. As previously reported (Charest-Morin et al., 2013), EGFP-MK was produced as the lysate of producer cells and diluted in the culture medium of receptor expressing cells for a 30 min-period of stimulation at 37 °C. The intact cells were prelabeled with an anti-myc tag monoclonal antibody conjugated with AlexaFluor 594 (clone 9E10, R&D Systems, dilution 1:100, 15-min incubation at 37 °C) to evidence myc-B_2_R expression at the cell surface and eventual subcellular translocation. Cells were photographed using an Olympus BX51 microscope coupled to a CoolSnap HQ digital camera (filters for GFP: excitation 460–500 nm, emission 510–560 nm; for AlexaFluor 594: excitation 525–555 nm, emission 600–660).

### Data analysis

Results are presented as means ± s.e.m. Radioligand binding data were fitted by nonlinear regression to a one-site competition equation using a least-square method (Prism 5.0; GraphPad Software Inc., San Diego, CA, USA) and IC_50_ values calculated from this procedure. The same computer program was used to draw concentration-effect curves (least square fitting of sigmoidal dose–response equation with variable slope) and to derive contractile EC_50_ values. Data describing baseline values of heart rate and mean arterial blood pressure and hypotensive responses to peptides in anesthetized rats were assessed by using one-way analysis of variance (ANOVA) followed by the Dunnett’s test (repeated comparison with a common control). The effects of drugs on the hypotensive responses to each dose of MK were assessed by using ANOVA followed by the Dunnett’s test. The effect of icatibant on hemodynamic parameters influenced by doses of BK or MK in rats instrumented for Doppler flow recording was tested using Student’s *t* test (Prism 5.0 software).

## Results

### Affinity of the N-terminally extended kinin MK for recombinant myc-B_2_Rs

**Figure 1:**
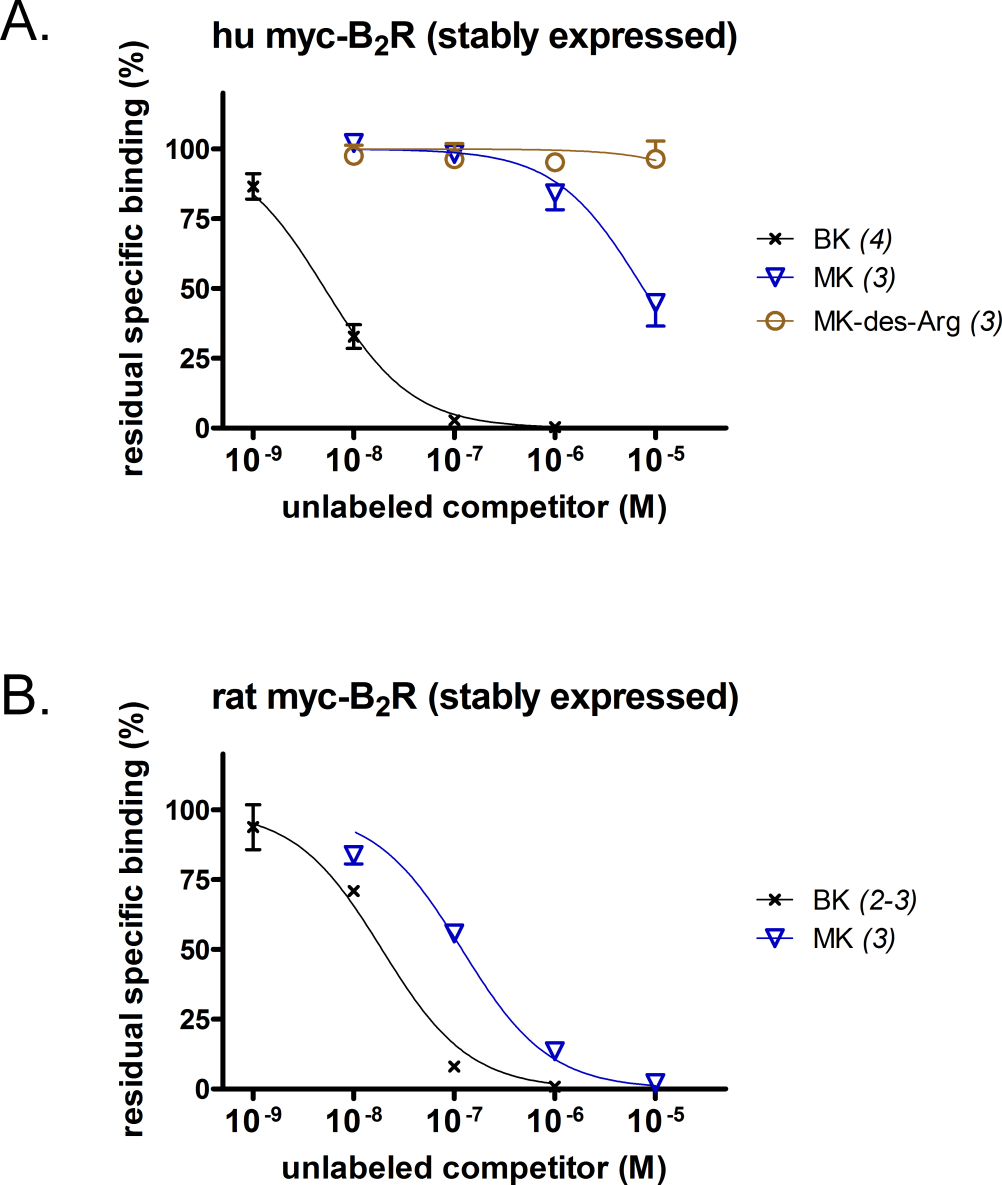
Pharmacology of N-terminally extended BK sequences in HEK 293a cells that stably express recombinant myc-B_2_R constructions. Competition of [^3^H]BK (3 nM) binding to receptors (A, human; B, rat) by a panel of unlabeled peptides. Results are expressed as the residual specific bindings (means ± SEM of the duplicate number of determinations indicated between parentheses). IC_50_ values and their 95% confidence limits are reported in Table 1.

A [^3^H]BK binding competition assay performed at water-ice temperature was exploited to determine the true receptor affinity of MK (Figs. 1A and 1B). This N-terminally prolonged BK sequence exhibited a very low affinity for the myc tagged B_2_R construction possessing the human sequence (Fig. 1A, relative potency *vs.* that of BK reported in Table 1). Thus, MK was ∼1,500-fold less potent than BK. At the rat recombinant myc-B_2_R, the radioligand binding competition assay indicated that MK had a lesser affinity than that of BK, but with narrower margin (6.2-fold; Fig. 1B). The relative potency of MK *vs.* BK was similar to the one established with an identical experiment performed with the rabbit B_2_R-GFP construction (Bawolak et al., 2012), thus substantiating a significant species-dependent affinity variation for MK across mammalian species. MK-des-Arg did not significantly displace [^3^H]BK binding to the human myc-B_2_R construction (Fig. 1A), consistent with the obligatory role of the C-terminal arginine residue of kinins for a good affinity at B_2_Rs (Leeb-Lundberg et al., 2005).

**Figure 2:**
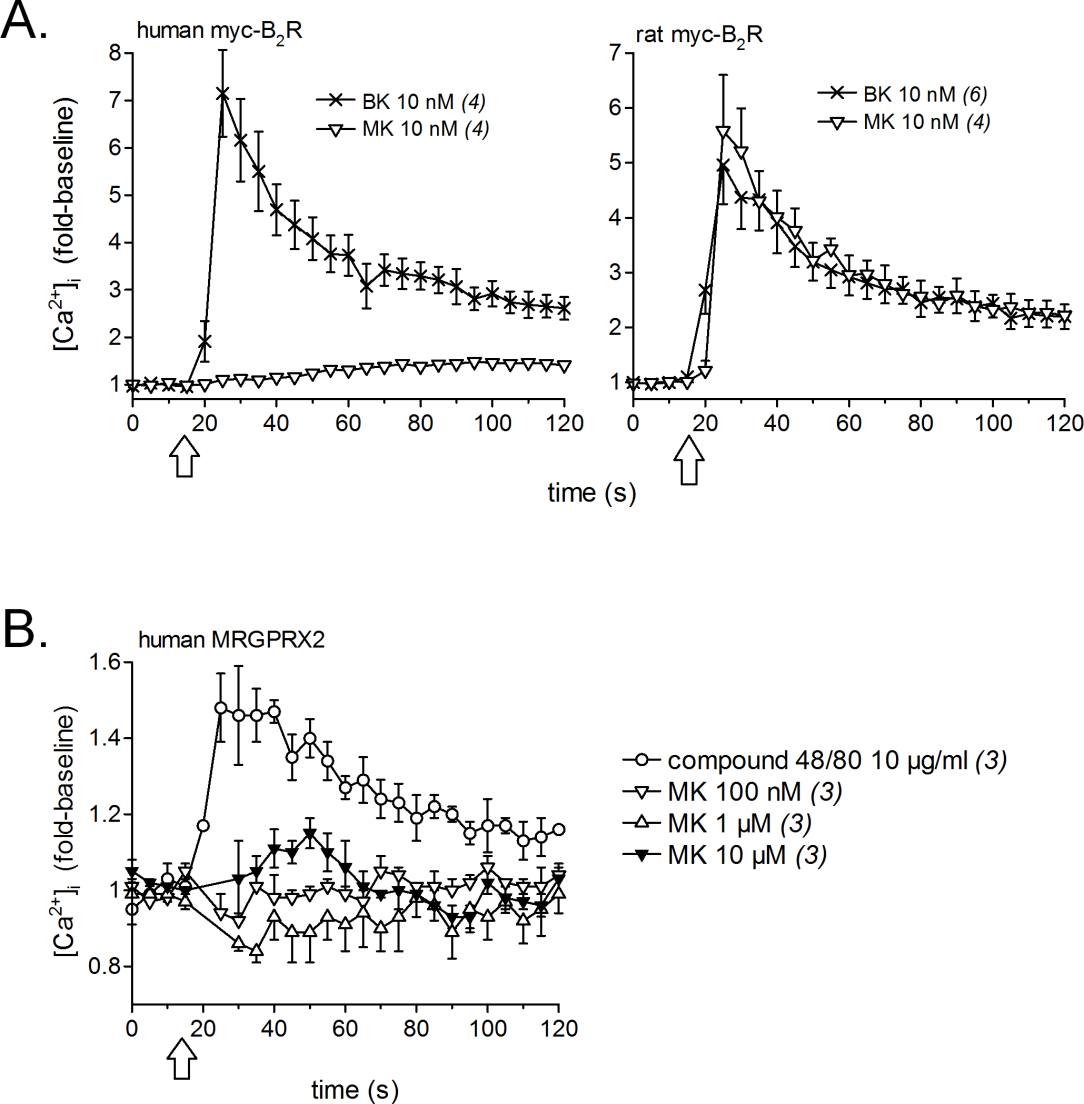
Pharmacology of MK in HEK 293a cells: calcium mobilization. (A) Effects on cells stably expressing myc-B_2_Rs from either species (positive control, BK). (B) Effect on cells transiently expressing human MRGPRX2 (positive control, compound 48/80). Tracings, obtained via Fura-2 fluorescence, represent calcium mobilization, in fold of basal values to show the time course of effects elicited by receptor ligands (conventionally applied at time 15 s, arrows). The number of determinations is indicated between parentheses.

Calcium signaling in HEK 293a cells stably expressing either human or rat myc-B_2_Rs was conducted with a constant agonist concentration (10 nM, Fig. 2A). A sizeable and immediate signal was elicited by BK in cells expressing receptors from either species; MK also induced a strong but slightly protracted response *via* the rat receptor, but was virtually inactive at the human myc-B_2_R. The mast cell receptor MRGPRX2 (human sequence), transiently expressed in HEK 293a cells, mediated calcium transients in response to compound 48/80 (10 µg/ml, positive control; McNeil et al., 2015), but MK was marginally active only at 10 µM at this receptor (Fig. 2B).

### Human umbilical vein contractility and LC–MS

**Figure 3:**
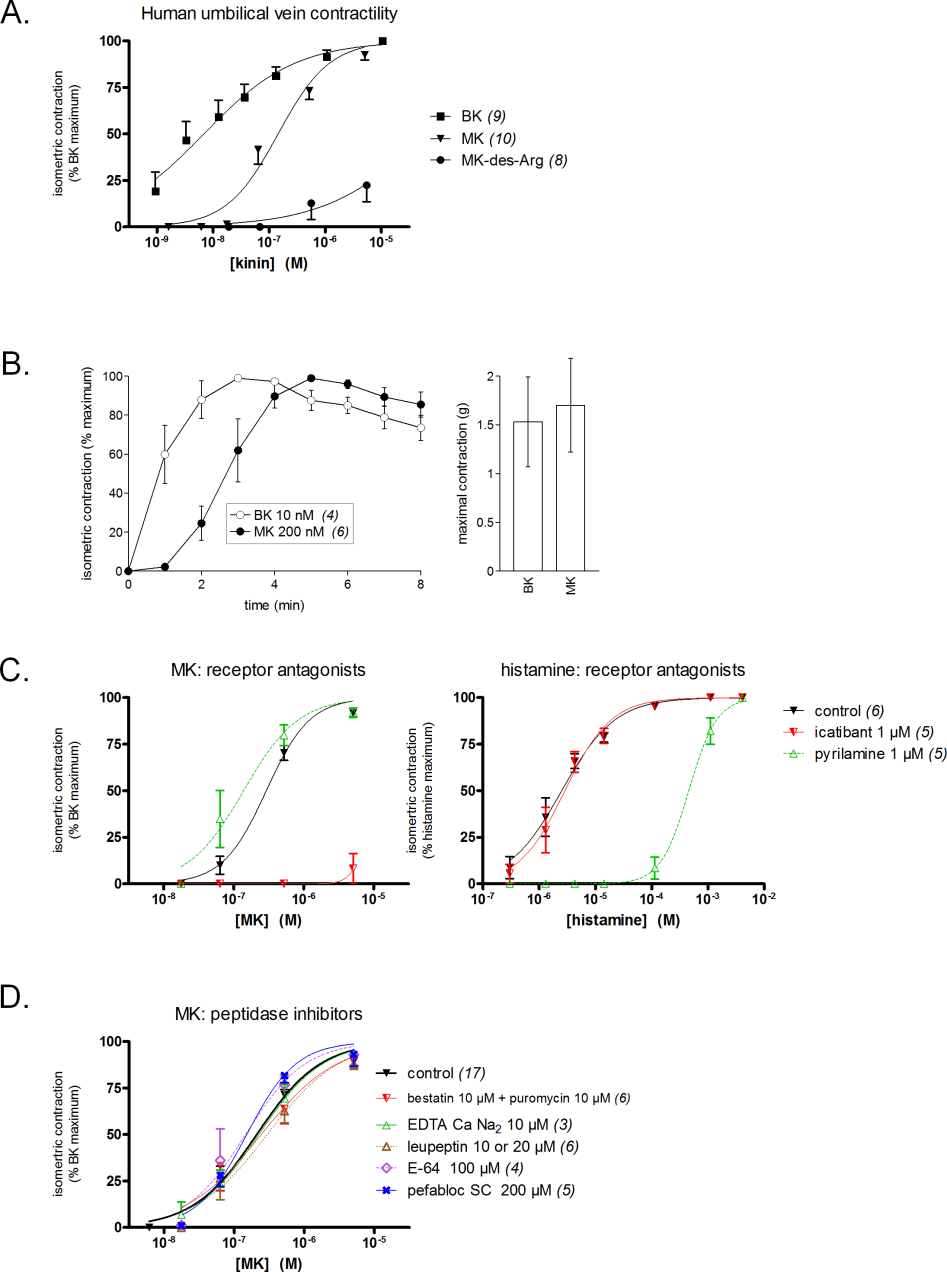
Contractility studies in the human isolated umbilical vein, a bioassay for the human B_2_Rs, applied to BK and to the N-terminally prolonged homolog MK. (A) Cumulative concentration-effect curves were constructed for the indicated peptides; for lower potency agonist MK, the maximal effect mediated by B_2_Rs was assessed by applying a maximal concentration of BK (9.4 µM) at the end of the curve construction. (B) Kinetics of the contraction induced by equipotent concentrations of BK and MK (concentrations as indicated). The similar amplitude of the maximal responses expressed in gram-weight has been verified. (C) Effect of receptor antagonists (applied 30 min before agonists) on the concentration-effect curves of MK and histamine. (D) Effect of protease/peptidase inhibitors (applied 30 min before MK) on the concentration-effect curve of MK. (C) and (D) are presented as panel (A). In all panels, values are means ± SEM of the number of replicates indicated between  parentheses.

The freshly isolated human umbilical vein is a contractile bioassay for the B_2_R exploited in several laboratories (Altura et al., 1972; Marceau et al., 1994; Marceau et al., 2010; Gobeil et al., 1996). BK elicits a response at subnanomolar concentrations in this preparation (Fig. 3A). Contrasting with the very low affinity of MK for the human B_2_R in the binding competition assay, this prolonged peptide was only ∼20-fold less potent than BK in the contractility assay (Fig. 3A, EC_50_ values reported in Table 1). This suggests a conversion of MK by attack at the N-terminus, BK being the shortest B_2_R agonist of high affinity (Leeb-Lundberg et al., 2005). However, MK-des-Arg has negligible effects in the contractility assay, in line with the binding assay.

Equipotent concentrations of kinins (approximately EC_50_ values, from Fig. 3A) were chosen to compare the contractile kinetics of the venous preparation (Fig. 3B). It was verified that BK and MK produced a similar contractile response expressed in gram-weight (Fig. 3B, right). However, the contraction induced by BK (10 nM) was rapidly developing and reaching a plateau, whereas MK (200 nM) induced a slowly developing response (Fig. 3B, left). These observations further support an indirect response of the N-terminally prolonged peptides on the human B_2_Rs. That the effect of MK on the umbilical vein is ultimately mediated by the BK B_2_Rs is shown by the profound inhibitory effect of icatibant (1 µM), an antagonist of this receptor (Fig. 3C). The H_1_R antagonist pyrilamine (1 µM) did not reduce the venous effect of MK, while profoundly and competitively inhibiting the contractile effect of histamine (Fig. 3C). Thus, histamine release does not contribute significantly to the contractile effect of MK in this preparation.

Whether the venous preparation at 37 °C has peptidase/protease activities that could produce shorter peptides with high receptor affinities from MK has been tested in different groups of tissues pre-treated with various inhibitors (Fig. 3D). The combination of aminopeptidase inhibitors, bestatin and puromycin (Hitzerd et al., 2014), had no effect on the apparent contractile potency of MK. The same applied to the metallopeptidase inhibitor EDTA calcium disodium, an agent that modulated the apparent potency of kinins in a previous study of the rabbit isolated aorta (Babiuk et al., 1982), and to the wide spectrum protease inhibitor, leupeptin (Zhang et al., 2005). The cysteine protease inhibitor E-64 and the serine protease inhibitor pefabloc SC also failed to change the concentration-effect curve of MK (Fig. 3D). In principle, this observation rules out the metabolic activation of MK by many serine proteases, including kallikreins. The dual ACE and neutral peptidase inhibitor omapatrilat also failed to modify the contractile effect of MK (data not shown).

**Figure 4:**
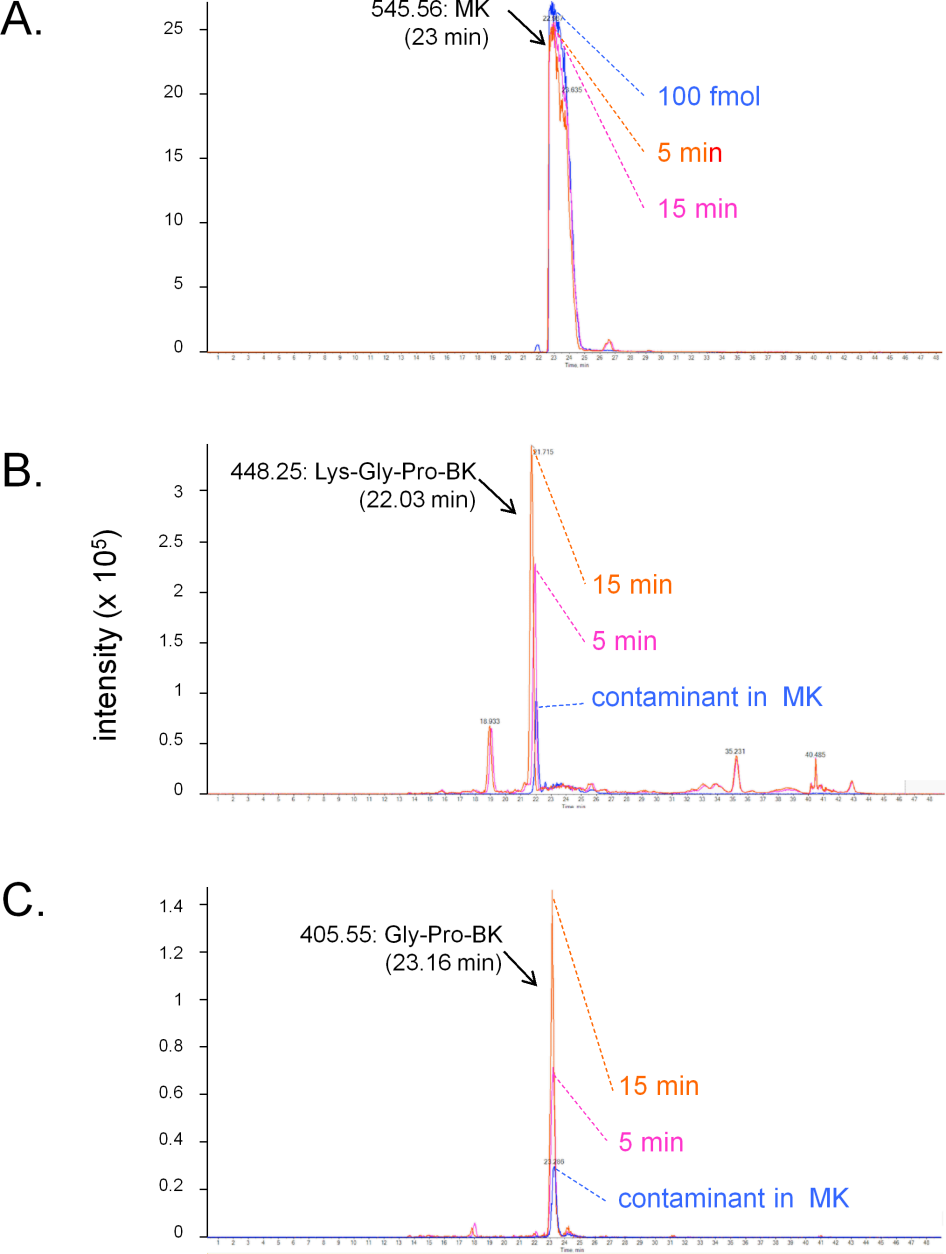
LC-MS determination of C-terminal fragments of MK dissolved in sterile Krebs solution and incubated in the presence of a ring of human umbilical vein at 37 °C for 5 or 15 min. Only the high resolution extracted ion chromatograms are shown; peptide identity was ascertained by MS and MS/MS. (A) MK (100 fmol) is consumed during the reaction. (B) Lys-Gly-Pro-BK is the major C-terminal metabolite. (C) Gly-Pro-BK is also a metabolite that accumulates. Typical results of duplicate determinations. Dotted lines indicate the summit of the indicated peaks.

The conversion of MK into shorter, high affinity kinin(s) in the human vein is a logical necessity due to the marginal affinity of MK for the human B_2_R and to the slow contraction kinetics of MK; however, individual proteases covered by the panel of inhibitors may not be kinetically limiting for this conversion. We addressed the possible generation of pharmacologically active C-terminal fragments of MK using LC-MS analysis of peptides extracted from Krebs solution containing MK (1 µM) and incubated with rings of umbilical veins (Fig. 4). Among the most intense ions, two C-terminal fragments were found. The release of Lys-Gly-Pro-BK and Gly-Pro-BK was evidenced in a time-dependent manner (Fig. 4; sequence confirmed by tandem mass spectroscopy, Fig. S1), supporting the time-dependent generation of BK-like peptides at the vicinity of tissue receptors. Surprisingly, the commercial peptide MK contained small amounts of both C-terminal fragments as contaminants, but the reaction with the tissue increased 3-4-fold the intensity of the corresponding ions. Abundant N-terminal fragments of MK were also identified, such as DLPKINRKGPRPP and DLPKINRKGPRPPGF (data not shown), supporting that multiple cleavage sites are present in the MK sequence under these conditions.

### Species-dependent variation of cell labeling with EGFP-MK

**Figure 5:**
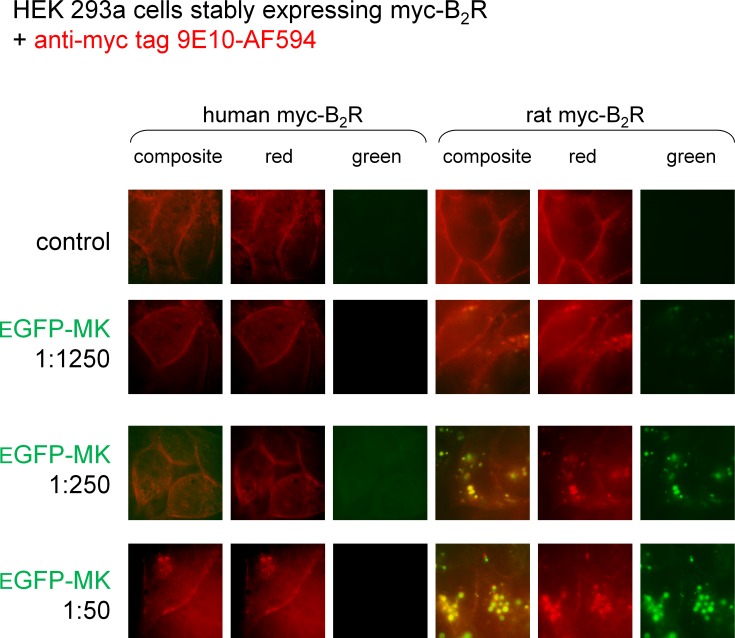
Staining of HEK 293a cells stably expressing a myc-B_2_R construction by EGFP-MK as a function of the mammalian species origin of the receptor sequence. Prior to the stimulation with EGFP-MK (30 min, 37 °C; producer cell lysate dilution as indicated), the cells were incubated *in vitro* with the AlexaFluor-594-conjugated anti-myc monoclonal antibody 9E10, providing a red marking of cell surface myc-B_2_Rs. Representative of two separate experiments. The sides of square fields measure 50 µm.

MK is the B_2_R-binding module in the fusion protein EGFP-MK (Charest-Morin et al., 2013). We reported that the protein is transported in endosomes by both the rabbit and human B_2_Rs (Charest-Morin et al., 2013; Charest-Morin & Marceau, 2016), but wished to run a side-by-side comparison of the rat and human B_2_R-mediated uptake as a function of the concentration of EFGP-MK (Fig. 5). In these experiments, myc-B_2_R possessing the human or rat receptor sequence, were prelabeled at the surface of live cells with anti-myc tag monoclonal antibodies conjugated to a red fluorophore. Then the intrinsically green fluorescent protein EGFP-MK was added for incubation at 37 °C. Results show that the fusion protein is more potent at the rat myc-B_2_R, which mediated the endocytosis of EGFP-MK along with extensively co-localized anti-myc antibodies, supporting the persistence of agonist-receptor complexes in endosomes at the examined time point. EGFP-MK has comparatively little effect at the human myc-B_2_R construction, except for initiating a partial endocytosis of labeled receptors at 1:50 dilution without significant green signal (Fig. 5). This response is comparable to that of the 1:1,250 dilution of EGFP-MK at the rat receptor.

### *In vivo* hemodynamic responses to MK

Baseline values for MAP and HR measured in the untreated control group or 15 min after i.v. pretreatment with enalaprilat, icatibant or pyrilamine are shown in Table 2. While no significant changes in basal values of MAP were noted between the groups, a slight but significant increase in basal HR was found between the enalaprilat pretreated groups and the control group (Table 2).

**Table 2:** Basal cardiovascular parameters in anesthetized, pre-treated rats.

Pre-treatment	Mean arterial blood pressure	Heart rate	*n*
Control	96.5 ± 2.5	351 ± 10	25
Enalaprilat 0.1 mg/kg	94.9 ± 4.6	417 ± 13^*^	6
Icatibant 25 µg/kg	90.3 ± 4.6	362 ± 28	6
Pyrilamine 5 mg/kg	87.4 ± 2.7	335 ± 6.1	16
ANOVA	*P* = 0.13	*P* = 0.009	

**Notes.**

* *P* < 0.01, Dunnett’s test *vs*. control.

**Figure 6:**
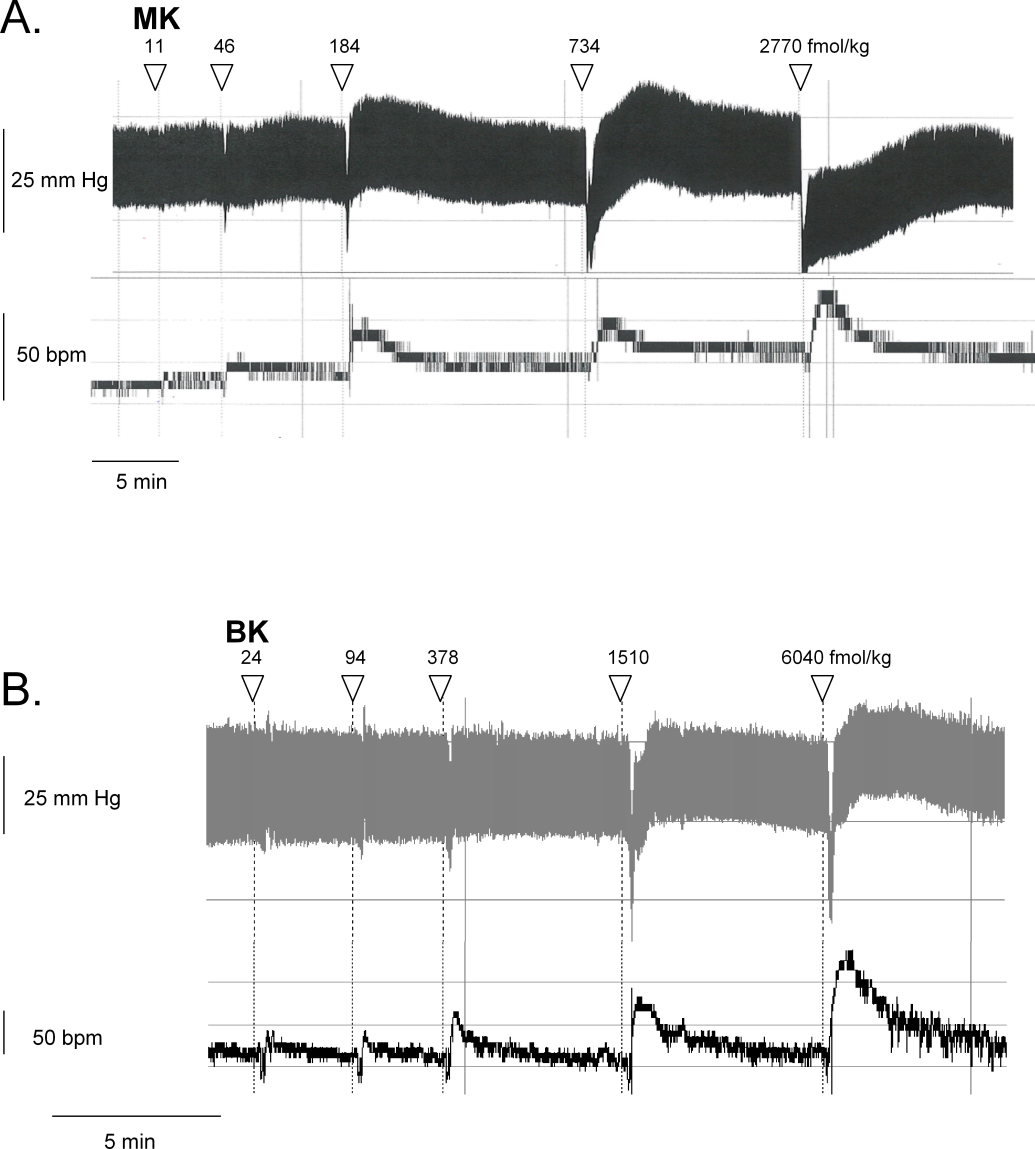
Hemodynamic responses to i.v. bolus injections of increasing doses of B_2_R agonists in anesthetized rats. Representative traces showing the dose-response effect of MK (A) or BK (B) on systemic blood pressure and heart rate. Doses of MK or BK peptides are expressed as fmol/kg to offset the wide difference of their molecular weight.

**Figure 7:**
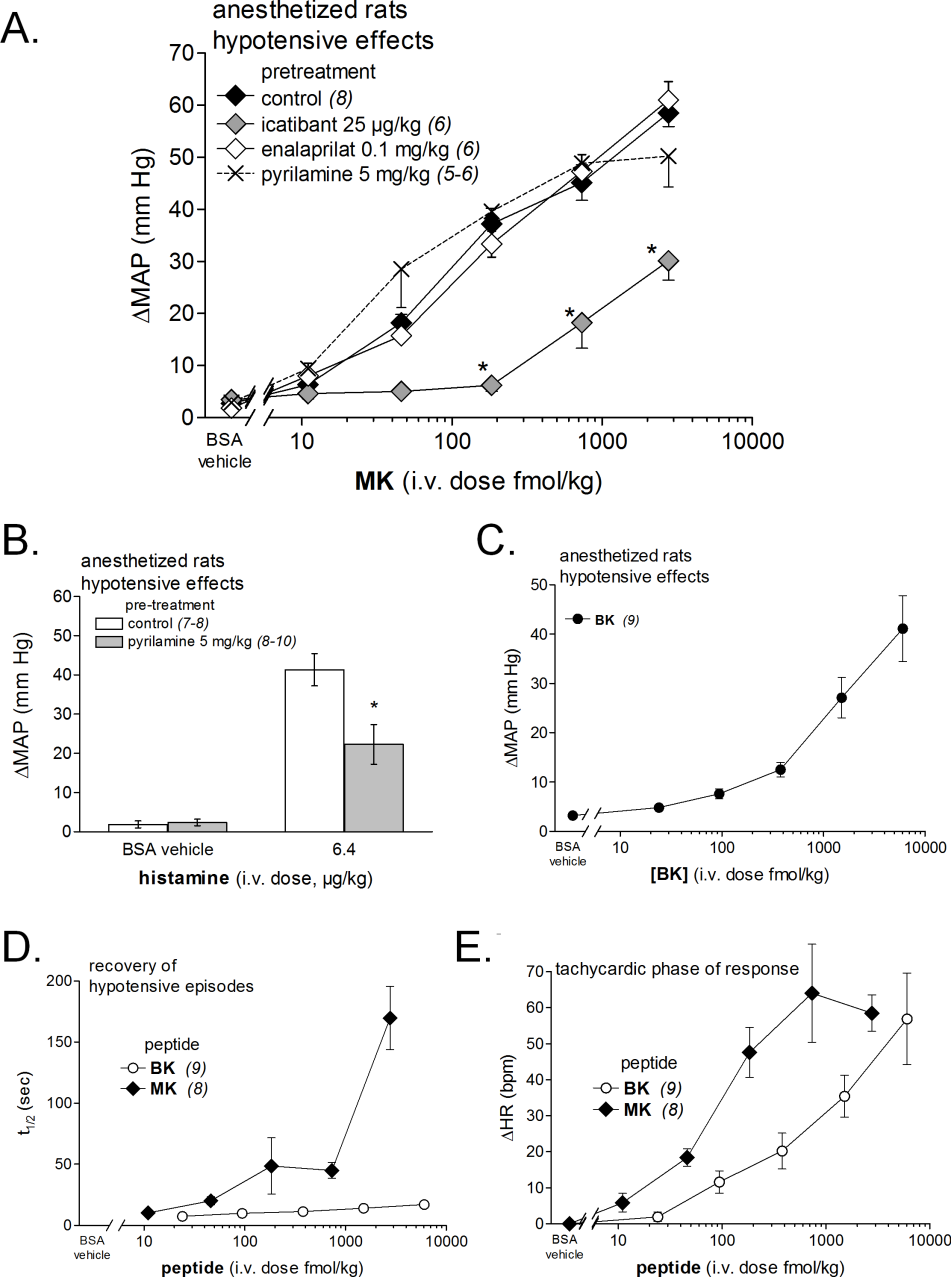
Hemodynamic responses to i.v. bolus injections of increasing doses of B_2_R agonists in anesthetized rats. (A) Dose-response curves for the hypotensive effect of MK in replicated experiments in separate groups of rats and effect of pretreatments with icatibant, enalaprilat or pyrilamine (given as i.v. boluses 15 min before starting the injections of MK) on the hypotensive effect of MK. Abscissa: dose (fmol/kg); ordinate: fall of mean arterial blood pressure (MAP; mm Hg). The effects of MK were significantly different from that of the saline-BSA vehicle at the four superior dose levels (ANOVA followed by Dunnett’s test, *P* < 0.01). *, *P* < 0.01: effect of pretreatment on MK hypotensive effects (Dunnett’s test that followed significant ANOVA for each MK dose). (B) Bar graph showing the validation of pyrilamine effect in anesthetized rats intravenously injected with histamine, as indicated. The pretreatment with pyrilamine (given as i.v. bolus 15 min before starting the injections of histamine) was made in a separate group of rats. The effect of histamine was significantly different from that of the saline-BSA vehicle (*, *P* < 0.01, Student’s *t* test). (C) Dose-response curves for the hypotensive effect of BK in replicated experiments in a separate group of rats. Presentation as in (A). The effects of BK were significantly different from that of the saline-BSA vehicle only at the two superior doses. (D) Recovery half-time for hypotensive episodes induced by the indicated peptides and reported in panels A and C. (E) Maximal tachycardic response during hypotensive episodes induced by the indicated peptides and reported in panels A and C. In all panels, values are means ± SEM shown by vertical lines and the number of determinations is indicated between parentheses.

We recently described the brief hypotensive responses associated with tachycardia to the i.v. injection of increasing doses of BK in anesthetized rats and the strong potentiation of the responses following pharmacologic ACE blockade (Jean et al., 2016a). Using the same methods, MK was consistently more potent than BK to produce hypotension (Figs. 6 and 7). Further, the hypotensive episodes elicited by MK lasted longer and the tachycardia was generally more intense than what was recorded in rats injected with increasing doses of BK (Figs.7D and 7E). In sharp contrast to the effects of i.v. BK (Jean et al., 2016a), MK was not significantly potentiated by pretreatment with the ACE inhibitor enalaprilat, but the effect of the prolonged peptide was abated by the B_2_R antagonist icatibant (Fig. 7A). Pyrilamine did not antagonize the effects of MK (Fig. 7A), and had no additional effect if combined with icatibant (data not shown). Pyrilamine, at the employed dose level, abated the hypotensive effect of histamine tested in separate groups of rats (Fig. 7B).

**Figure 8:**
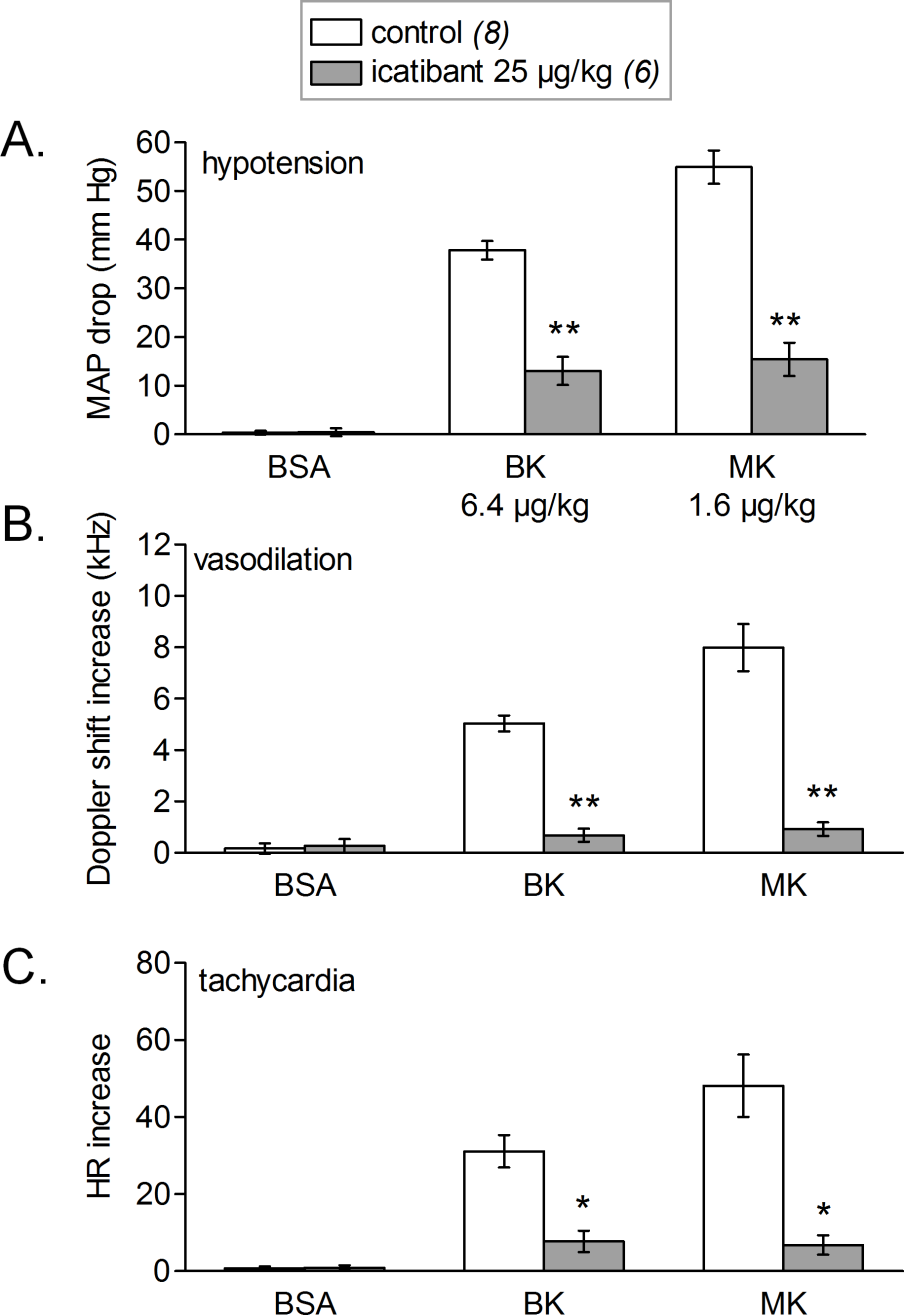
Simultaneous changes in mean arterial pressure (MAP, mm Hg, fall, (A)), mean hindquarter Doppler shift signal (kHz, rise, (B)) and heart rate (HR, beats per minute, tachycardic phase, panel (C)). Responses were elicited by i.v. injections of saline-BSA vehicle, BK or MK boluses in rats treated or not with icatibant (all peptide dosages indicated). Values are means ± SEM shown by vertical lines and the number of determinations is indicated between parentheses. Icatibant significantly abated all types of responses to the 2 kinins (Student’s *t* test, *, *P* < 0.01; **, *P* < 10^−4^).

Figure 8 shows the simultaneous changes in mean arterial blood pressure, mean hindquarter Doppler shift signal and heart rate induced by i.v. injections of vehicle, BK and MK administered at doses producing a sizeable hypotensive effect of 40–55 mmHg. We found that, for both tested agonist, the observed hypotensive responses were accompanied with concomitant increases in mean Doppler shift signals and heart rate when compared with vehicle values. The increases in mean hindquarter Doppler shift might reflect a reduction in vascular resistance (vasodilation) redistributing flow to the hindlimb muscles, as previously shown in conscious rats i.v. injected with BK (Bachelard et al., 1992). Interestingly, the cardiovascular responses elicited by BK or MK were all significantly abated by systemic pretreatment of rats with icatibant, suggesting actions mediated by cardiovascular B_2_Rs.

## Discussion

The proposed use of the MK sequence in the design of fusion proteins intended to bind to the BK B_2_R (Charest-Morin et al., 2013) is based on a docking model where the C-terminus of receptor-bound BK is close to the extracellular fluid interface (Leeb-Lundberg et al., 2005), thus leading to toleration of N-terminal sequence extension. The development of diagnostic or therapeutic applications of B_2_R-binding fusion proteins required that we formally verified the affinity of MK at the human and rat B_2_R to support future clinical and pre-clinical investigations. Our initial report on MK pharmacology included a [^3^H]BK binding competition assay involving the construction B_2_R-GFP that is based on a rabbit receptor sequence (Bawolak et al., 2012). The results were very similar to those obtained with the rat B_2_R (Fig. 1B) with a loss of affinity of approximately 1 log unit *vs.* BK. The moderate decrease of potency of MK *vs.* BK in the umbilical vein contractility assay, replicated in the present experiments (Fig. 3A), seemed at the time reasonably in line with the radioligand binding study conducted with the rabbit B_2_R. Unexpectedly, the affinity of MK for the human B_2_R has been found to be very low when compared to that for the rat B_2_R (Figs. 1A and 2A). Accordingly, the fusion protein EGFP-MK has a low potency to label the human B_2_Rs, whereas it was very active at the rat B_2_R (Fig. 5). These findings contrast with the fair potency of MK in the human venous contractility assay (Fig. 3).

The isolated umbilical vein has a low intrinsic sensitivity to endothelium-dependent vasodilation owing to its >20 layers of B_2_R-expressing smooth muscle cells (Koumbadinga et al., 2010; Jean et al., 2016b). The umbilical vein contractility assay has been found suitable to evidence the metabolic activation of BK prodrugs prolonged at their C-terminus by vascular ACE and arginine-carboxypeptidases (Gera et al., 2011; Charest-Morin et al., 2014): these peptides, such as BK-His-Leu, BK-Arg, and Met-Lys-BK-Ser-Ser, exhibited a decreased apparent potency in the presence of specific peptidase inhibitors that did not affect BK EC_50_, supporting *in situ* regeneration of BK, Lys-BK or Met-Lys BK (the latter two high affinity agonists only from Met-Lys-BK-Ser-Ser). We reasoned that the low affinity peptides MK must also be activated in the venous preparation, producing active fragments not shorter than BK because any BK fragment has insignificant affinity for the B_2_R (Leeb-Lundberg et al., 2005). In addition, this conversion does not seem to operate in HEK 293a cells that express human B_2_Rs because MK produced no consistent calcium transients in these cells (Fig. 2A) that also only modestly internalized EGFP-MK (Fig. 5). Therefore, the metabolic activation of MK in the umbilical vein, also supported by its slow contractile kinetics (Fig. 3B), must derive from one or more peptidase activities specific for vascular tissue or, perhaps, ones released from lysed cells that inevitably are present in the venous rings. Our screening of peptidase/protease inhibitors has not revealed a kinetically limiting step for the metabolic activation of MK, suggesting perhaps that more than one pathway are operating over time in the system. This is further supported by the semi-quantitative application of LC-MS where multiple cleavage products of MK, including C-terminal fragments slightly larger than BK, were found to be generated following incubation with venous tissues (Fig.  4).

MRGPRX2 has been recently described as a promiscuous receptor for cationic polymers and drugs that produce direct mast cell degranulation (McNeil et al., 2015). Interestingly, the B_2_R antagonist icatibant itself activates MRGPRX2 but at concentration levels that may be 5 to 6 orders of magnitude larger than that effectively antagonizing the BK B_2_R (McNeil et al., 2015). MK has minimal activity at human MRGPRX2 starting at 10 µM (Fig. 2B), but may not exceed 100 nM in the plasma after injection at the highest tested dose *in vivo*. The contractile effect of MK on the human umbilical vein and its hypotensive effect in rats are unaffected by the H_1_R antagonist pyrilamine, ruling out a significant mediation by histamine. The vasodilator response to the mast cell activator compound 48/80 is mediated by histamine released from mast cells in the rat mesenteric vascular bed and abated by a H_1_R antagonist (Jin et al., 2016). On the other hand, the human umbilical artery does not contain preformed histamine or mast cells (Marceau et al., 1990), and this may apply to the umbilical vein as well. Altogether, results indicate that the vascular effects of MK are B_2_R-mediated, but not likely to be directly mediated by mast cell activation. The BK B_1_ receptor (B_1_R) is not significantly expressed in the cardiovascular system of the healthy rat (Jean et al., 2016a) or in the umbilical vein contractility assay unless special procedures are applied to upregulate the receptor expression (Jean et al., 2016b). Thus, mediation of any pharmacologic effect of MK by the B_1_R receptor is unlikely in the reported results. This is corroborated by the inhibition of MK effects by the selective B_2_R antagonist icatibant in both systems (Figs. 3C and 7A). Further, EGFP-MK does not label the recombinant B_1_R in microscopic observations (Charest-Morin et al., 2013). Finally, MK-des-Arg has a very low affinity for the human B_1_R in a [^3^H]Lys-des-Arg^9^-BK binding displacement assay (Charest-Morin & Marceau, 2016), suggesting that MK cannot be converted into an effective B_1_R agonist *via* reaction with hypothetical carboxypeptidases, including ACE2. Any effect of MK on AT_1_ receptor for angiotensin, represented in the human umbilical vein and mediating a contractile response to angiotensin II (Altura et al., 1972; Kalenga et al., 1991), is ruled out by the inhibition of MK-induced contractile effects by icatibant. Interaction of MK with other molecules belonging to the renin-angiotensin system, such as AT_2_ or mas receptors, remains undefined but unlikely considering that MK is a BK  homologue.

Strong species-specific responses to a toxin-derived peptide such as MK is plausible (Xi et al., 2015) and its empirically discovered prodrug status in human systems suggests important differences of the topography of the interface between the B_2_R and extracellular fluid across mammalian species. Unlike other BK homologues found in venoms, such as *Polistes* kinin, MK is not an efficient mast cell activator but its metabolic resilience may contribute to its toxicity in relevant predator species of *B. maxima*. MK is a pharmacophore module that confers affinity for the rat B_2_R, as in the EGFP-MK fusion protein, but not efficiently for the human form of the B_2_R. Further applications of MK, e.g., as a vasodilator drug or as a module for the construction of fusion proteins, will be limited by its species-specific pharmacology.

##  Supplemental Information

10.7717/peerj.2911/supp-1 Figure S1 Confirmation of the sequence of 2 C-terminal fragments of MK identified in samples of MK incubated for 15 min in the presence of a human umbilical vein ring *via* tandem mass spectroscopy (LC-MS/MS) The found N-terminal fragments of “n” residues (*b*_*n*_, in red) or of C-terminal ones (*y*_*n*_, in blue) of the postulated MK fragments are indicated along the mass/charge scale, thus confirming the identity of the parent peptides Gly-Pro-BK (A) and Lys-Gly-Pro-BK (B).

10.7717/peerj.2911/supp-2 Data S1 Individual replicates for each averaged data point in Figs. 1, 2, 3, 7 and 8.

## Additional Information and Declarations

### Data Availability

The following information was supplied regarding data availability: The research in this article did not generate, collect or analyse any raw data or code.
