# Supplementary figures and images for: Species-specific pharmacology of maximakinin, an amphibian homologue of bradykinin: putative prodrug activity at the human B2 receptor and peptidase resistance in rats
Source: PeerJ. 2017 Jan 18;5:e2911. doi: 10.7717/peerj.2911 (PMC5248581; doi:10.7717/peerj.2911)

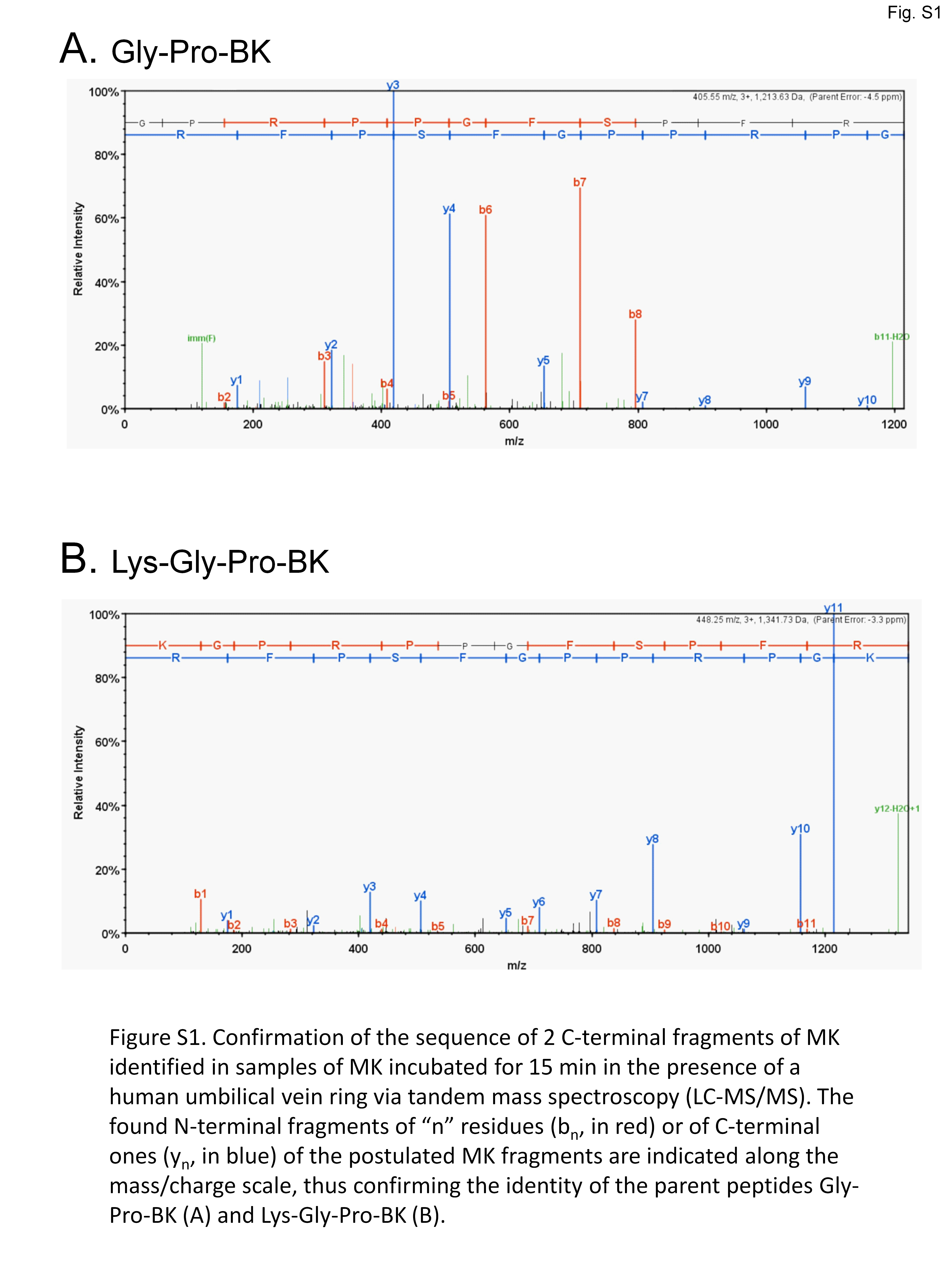

Supplement: Figure S1 — The found N-terminal fragments of “n” residues (bn, in red) or of C-terminal ones (yn, in blue) of the postulated MK fragments are indicated along the mass/charge scale, thus confirming the identity of the parent peptides Gly-Pro-BK (A) and Lys-Gly-Pro-BK (B). [file peerj-05-2911-s001.png]
