# Supplementary material for: Species-specific pharmacology of maximakinin, an amphibian homologue of bradykinin: putative prodrug activity at the human B2 receptor and peptidase resistance in rats
Source: PeerJ. 2017 Jan 18;5:e2911. doi: 10.7717/peerj.2911 (PMC5248581; doi:10.7717/peerj.2911)
Supplement: Data S1 [file peerj-05-2911-s002.pdf]

Raw numerical data

Fig. 1A : human B2R

Cold competing peptide→	BK				MK			MK-des-Arg		
Cold competitor concentration↓										
1 nM	99.24	86.44	82.74	77.98						
10 nM	44.19	28.52	24.58	33.80	106.06	102.09	98.39	90.20	102.84	99.66
100 nM	3.45	2.31	2.44	2.76	100.44	92.52	101.79	85.37	103.86	99.82
1 µM	.96	.29	.09	.05	88.96	91.24	72.37	95.93	99.73	90.19
10 µM					61.30	37.41	35.73	85.10	96.75	107.47

Fig. 1B : rat B2R

Cold competing peptide→	BK			MK		
Cold competitor concentration↓						
1 nM	85.75	101.90				
10 nM	71.78	70.20		87.22	86.86	77.47
100 nM	9.20	6.81		57.60	59.09	50.58
1 µM	0.00	0.27	2.13	13.51	13.19	13.91
10 µM				2.13	2.38	1.81

Fig. 2A, 2B : human, rat B2R

species→	human								rat									
agonist→	BK				MK				BK						MK			
time (s) ↓																		
0	.99	.98	.98	.92	.93	1.01	1.05	1.00	1.05	.95	.91	1.08	.97	1.01	.95	.91	.97	1.08
5	.98	1.04	1.05	1.04	1.02	.96	1.00	.93	.99	.97	.91	.89	1.04	.99	1.08	.91	1.0	.95
10	1.03	.98	.98	.98	1.05	1.03	.98	1.11	.96	1.08	1.01	1.08	.91	.97	.97	1.08	.99	1.00
15	.86	.95	.99	1.05	.96	1.02	.97	.97	1.20	1.09	1.17	.96	1.05	.99	.93	1.10	1.02	.97
20	1.09	2.26	2.95	1.32	1.11	.98	.96	1.00	4.01	2.31	3.35	2.36	1.79	1.94	.95	.96	1.71	1.23
25	6.02	5.21	9.16	8.2	1.12	1.05	1.04	1.16	6.34	4.96	4.70	7.50	3.13	3.14	5.94	6.19	7.51	2.71
30	5.15	4.27	7.97	7.24	1.18	1.11	1.08	1.11	5.61	5.11	5.23	7.71	3.12	2.80	5.79	5.51	6.57	2.95
35	4.34	3.84	7.33	6.5	1.085	1.18	1.02	1.09	4.60	4.24	5.14	6.34	2.67	2.99	4.35	4.82	5.27	2.76
40	3.92	3.62	5.76	5.46	1.21	1.14	1.07	1.18	4.48	3.73	3.92	6.24	2.45	2.63	4.40	4.59	4.48	2.56
45	3.85	3.24	5.52	4.86	1.24	1.18	1.11	1.09	3.91	3.33	3.64	4.95	2.39	2.67	4.01	4.18	4.31	2.54
50	3.58	3.13	5.14	4.45	1.38	1.22	1.15	1.19	3.85	3.80	3.21	3.94	2.09	2.22	3.34	3.49	3.75	2.31
55	3.19	3.02	4.64	4.19	1.39	1.28	1.35	1.24	3.39	3.45	2.98	4.15	1.88	2.45	3.22	3.26	3.82	2.37
60	3.08	2.91	4.46	4.47	1.32	1.33	1.27	1.29	3.02	3.10	3.24	4.17	1.82	2.17	3.25	3.04	3.57	1.96
65	3.13	2.81	4.31	2.07	1.43	1.3	1.24	1.45	3.34	3.19	3.21	3.36	1.99	1.79	3.11	3.14	3.40	2.24
70	2.85	2.85	4.12	3.84	1.48	1.36	1.26	1.42	3.15	2.66	2.92	2.53	1.90	2.03	3.22	3.00	3.15	1.77
75	3.08	2.65	4.15	3.46	1.47	1.54	1.29	1.46	3.26	2.72	2.93	3.50	1.79	2.04	2.93	2.52	3.19	1.79
80	3.15	2.62	4.09	3.39	1.31	1.37	1.36	1.46	2.89	2.73	2.42	3.26	1.66	1.71	2.78	2.60	3.10	1.75
85	2.89	2.67	3.91	3.38	1.49	1.52	1.32	1.39	3.06	2.71	2.85	3.29	1.66	1.71	2.63	2.33	3.11	1.70
90	2.52	2.48	4.08	3.19	1.46	1.44	1.37	1.48	2.96	2.81	2.64	3.26	1.66	1.80	2.59	2.72	3.26	1.76
95	2.59	2.44	3.52	2.69	1.49	1.61	1.24	1.59	3.01	2.77	2.25	2.98	1.61	1.64	2.44	2.38	3.03	1.72
100	2.73	2.31	3.57	3.07	1.47	1.52	1.39	1.47	3.08	2.52	2.59	3.07	1.55	1.80	2.28	2.70	2.71	1.58
105	2.45	2.29	3.33	2.88	1.49	1.54	1.35	1.39	2.39	2.31	2.26	2.82	1.50	1.72	2.50	2.54	2.78	1.67
110	2.31	2.16	3.41	2.86	1.48	1.55	1.32	1.50	2.82	2.40	2.24	2.91	1.46	1.69	2.28	2.63	2.57	1.61
115	2.41	2.18	3.31	2.69	1.57	1.45	1.39	1.35	2.73	2.45	2.32	2.62	1.52	1.64	2.56	2.28	2.58	1.65
120	2.39	2.21	3.29	2.56	1.38	1.5	1.36	1.41	2.63	2.10	2.56	2.82	1.43	1.66	2.37	2.36	2.50	1.63

Fig. 2B : MRGPRX2

agonist→	compound 48/80			MK 100 nM			MK 1 µM			MK 10 µM		
time (s) ↓												
0	1.00	.98	.88	1.04	1.03	.97	.97	.99	1.01	1.01	1.10	1.04
5	.99	.99	.99	.98	.92	1.00	1.00	.96	1.01	1.00	1.03	1.02
10	.96	1.05	1.08	1.04	.95	.96	1.02	.94	1.01	.99	.98	1.05
15	--	.99	1.03	1.06	1.08	1.00	.97	.99	.95	.96	.98	1.05
20	1.17	--	--	--	--	--	---	--	--	.71	--	--
25	1.36	1.65	1.42	.84	1.03	.94	.77	.85	.83	.77	.98	1.00
30	1.34	1.71	1.33	.88	.95	.93	.83	.90	.84	.85	1.17	1.08
35	1.34	1.48	1.57	.93	1.02	1.07	.81	.90	.81	.98	1.13	1.05
40	1.35	1.56	1.50	.88	.98	1.09	.86	1.04	.88	1.01	1.19	1.14
45	1.29	1.48	1.40	.94	.98	1.02	.88	1.04	.76	1.04	1.15	1.12
50	1.32	1.41	1.29	.96	.98	1.04	.93	.99	.74	1.07	1.22	1.17
55	1.25	1.42	1.35	1.01	.99	1.03	.83	1.02	.95	1.01	1.13	1.17
60	1.23	1.32	1.26	.96	1.01	1.00	.86	1.04	.83	.94	1.05	1.19
65	1.19	1.30	1.38	.93	1.02	.95	.88	1.11	.83	.94	1.04	1.05
70	1.22	1.16	1.33	.98	1.06	1.10	.83	1.02	.85	.97	1.00	.99
75	1.18	1.18	1.32	.99	1.06	1.06	.85	1.11	.84	.91	1.01	1.09
80	1.09	1.30	1.17	1.01	1.01	1.00	.94	1.07	.92	.99	.98	1.00
85	1.18	1.16	1.27	.95	1.01	1.08	.88	1.00	.99	.89	1.04	.96
90	1.16	1.21	1.23	.95	1.03	1.03	.90	1.00	.77	.87	.93	.99
95	1.14	1.10	1.21	1.05	1.04	.98	.85	1.07	.92	.88	.94	.98
100	1.08	1.13	1.31	1.01	1.06	1.11	.88	1.06	.86	.96	1.03	1.06
105	1.15	1.15	1.20	1.03	.99	1.03	.92	1.02	.96	.93	1.03	.99
110	1.05	1.13	1.21	.97	1.01	1.05	.92	1.03	.81	.94	.99	.99
115	1.10	1.08	1.24	.92	1.09	1.01	.86	1.10	.90	.90	.97	1.00
120	1.15	1.16	1.17	1.06	1.00	1.07	.92	1.08	.96	.93	1.90	1.03

Fig. 3A

**BK**										
Replicate no. →	1	2	3	4	5	6	7	8	9	10
↓Log [molar conc.]										
-9.030	73.500	15.5	0.0	0.8	1.0	8.5	1.2	71.4	1.5	
-8.480	73.500	20.7	3.8	40.0	39.8	14.1	79.3	82.1	67.2	
-7.900	85.700	37.9	21.0	45.0	69.4	25.0	79.3	92.9	76.3	
-7.440	89.800	51.7	34.2	61.7	79.6	51.6	81.9	96.4	80.9	
-6.880	91.800	62.0	53.3	81.6	85.7	79.7	89.2	100.0	87.8	
-5.970	100.000	69.0	83.8	98.3	100.0	79.7	96.4	100.0	96.2	
-4.980	100.000	100.0	100.0	100.0	100.0	100.0	100.0	100.0	100.0	
**MK**										
-8.793	0.00	0.0	0.0	0.0	0.0	0.0	0.0	0.0	0.0	0.0
-8.210	0.00	0.0	0.0	0.0	0.0	0.0	0.0	0.0	0.0	0.0
-7.752	6.50	0.0	0.0	5.5	1.1	0.6	1.0	0.0	0.0	0.0
-7.197	25.80	30.0	20.8	18.2	68.9	71.7	80.2	9.5	47.1	43.9
-6.282	54.80	70.0	77.6	43.6	88.9	86.2	88.3	81.6	70.6	70.7
-5.292	93.50	95.0	95.5	69.1	97.8	96.2	97.5	98.0	88.2	95.1
**MK-des-Arg**										
-7.719	0.0	0.0	0.0	0.0	0.0	0.0	0.0	0.0		
-7.165	0.0	0.0	0.0	0.0	0.0	0.0	0.0	0.0		
-6.249	71.3	10.4	0.0	20.7	0.0	0.0	0.0	0.0		
-5.259	71.3	45.8	15.7	20.7	0.0	1.1	22.2	2.6		

Fig. 3B: left, time course

agonist→	BK				MK					
time (min) ↓										
0	0	0	0	0	0	0	0	0	0	0
1	66.1	15.9	79.2	78.0	1.8	1.7	3.3	0	4.8	1.8
2	98.3	59.1	95.8	98.3	39.9	10.2	6.7	2	47.6	43.6
3	100	95.5	100	100	98.8	49.2	20	14	100	89.1
4	98.3	100	95.8	94.8	100	100	73.3	100	97.6	96.4
5	96.6	95.5	83.3	74.6	97.6	100	100	100	97.6	98.2
6	94.9	88.6	83.3	72.9	96.4	85.6	100	100	95.2	98.2
7	91.5	84.1	75	64.4	9.5	71.2	80	100	94.0	100
8	88.1	77.3	7.8	57.6	88.1	55.9	80	98	94.0	96.4

Fig. 3B, right, E_{max}

agonist→	BK				MK					
E_{max} (g)	2.89	1.29	1.03	.92	3.27	1.37	2.13	1.14	.91	1.19

Fig. 3C : left, MK: receptor antagonists

control						
Replicate no. →	1	2	3	4	5	6
↓Log [molar conc.]						
-7.752	0.000	0.0	0.0	0.0	0.0	0.0
-7.197	0.700	13.3	31.6	10.7	2.5	0.9
-6.282	75.400	75.0	78.9	68.0	51.9	72.4
-5.292	94.200	95.3	96.1	85.3	91.3	87.9
**icatibant**						
-7.752	0.00	0.0	0.0	0.0	0.0	
-7.197	0.00	0.0	0.0	0.0	0.0	
-6.282	0.00	0.0	0.0	0.0	0.0	
-5.292	0.00	0.0	0.0	0.0	40.5	
**pyrilamine**						
-7.752	0.0	0.0	0.0	0.0	1.1	
-7.197	2.7	25.3	66.7	4.5	75.0	
-6.282	83.6	79.1	87.5	59.1	89.8	
-5.292	88.4	94.5	95.1	84.1	97.7	

Fig. 3C : right, histamine

**control**						
Replicate no. →	1	2	3	4	5	6
↓Log [molar conc.]						
-6.523	0.800	1.0	37.5	10.4	2.9	0.0
-5.886	45.300	28.8	53.1	15.1	70.6	2.0
-5.367	65.600	57.6	71.9	58.4	82.4	60.0
-4.845	82.800	73.1	81.3	71.7	88.2	75.0
-3.942	97.700	94.2	90.6	94.3	100.0	94.0
-2.953	100.000	100.0	100.0	100.0	100.0	100.0
-2.386	100.000	100.0	100.0	100.0	100.0	100.0
**icatibant**						
-6.523	1.30	0.6	0.0	0.0	25.6	
-5.886	35.80	2.9	2.0	36.1	67.4	
-5.367	76.90	62.7	58.8	50.0	79.1	
-4.845	89.70	76.2	70.6	72.2	88.3	
-3.942	97.40	95.3	94.1	94.4	97.7	
-2.953	100.00	100.0	100.0	100.0	100.0	
-2.386	100.00	100.0	100.0	100.0	100.0	
**pyrilamine**						
-6.523	0.0	0.0	0.0	0.0	0.0	
-5.886	0.0	0.0	0.0	0.0	0.0	
-5.367	0.0	0.0	0.0	0.0	0.0	
-4.845	0.0	0.0	0.0	0.0	0.0	
-3.942	0.0	0.0	0.0	30.0	12.5	
-2.953	67.3	81.8	95.5	100.0	65.6	
-2.386	100.0	100.0	100.0	100.0	100.0	

Fig. 3D: MK, peptidase inhibitors

Replicate no. →	1	2	3	4	5	6	7	8	9	10	11	12	13	14	15	16	17
↓Log [molar conc.]																	
**control**																	
-7.752	1.700	0.0	0.0	0.0	0.0	0.0	0.0	12.5	0.0	0.0	0.000	0.000	0.000	0.000	0.000	0.000	3.000
-7.197	46.700	36.8	53.8	14.7	24.6	9.1	16.2	12.5	43.9	34.0	23.700	29.600	0.000	60.800	2.500	1.800	64.200
-6.282	85.000	76.3	73.8	68.1	64.6	53.2	77.4	75.0	65.9	70.0	57.900	77.800	45.600	80.300	70.400	82.100	91.000
-5.292	90.000	94.7	90.8	88.6	86.2	81.8	93.5	92.5	92.7	82.0	84.200	88.900	82.500	94.100	93.400	80.400	97.800
Replicate no. →	1	2	3	4	5	6											
**bestatin + puromycin**																	
-7.752	0.00	0.0	0.0	1.9	0.0	0.0											
-7.197	0.00	12.5	32.5	61.5	83.0	9.4											
-6.282	40.00	43.8	75.0	84.6	83.0	56.3											
-5.292	80.00	93.8	90.0	92.3	94.6	85.9											
**EDTA**																	
-7.752	20.6	0.0	0.0														
-7.197	33.0	28.8	8.4														
-6.282	84.5	63.0	61.1														
-5.292	94.8	91.8	93.4														
**leupeptin**																	
-7.752	0.0	0.0	0.0	0.0	0.0	0.0											
-7.197	20.5		0.0	48.4	5.4	58.5											
-6.282	56.4	54.7	38.8	76.9	73.2	75.6											
-5.292	82.1	92.6	76.1	90.1	92.0	95.1											
**E-64**																	
-7.752	0.0	0.0	4.8	0.0													
-7.197	20.0	81.3	40.5	2.2													

-6.282	66.7	88.8	81.0	71.0	
-5.292	90.0	96.3	95.2	89.9	
**Pefabloc SC**					
-7.752	1.7	0.0	0.0	3.0	0.0
-7.197	22.0	21.7	30.1	32.8	34.1
-6.282	86.4	78.3	79.5	79.1	85.3
-5.292	96.6	93.5	82.2	95.5	97.7

Fig. 7A: dose-response curves: ΔMAP, MK

Dose fmol/kg→	0	11	46	184	734	2770
**control**	4,8	7,3	21,4	36,8	48,9	62,3
	4,6	7,9	15,2	34,0	48,1	60,6
	1,9	2,0	12,6	23,0	34,1	51,9
	0,8	5,3	13,5	33,1	37,7	52,0
	3,0	5,7	25,5	48,8	49,1	69,8
	2,7	8,3	16,7	47,0	35,7	47,1
	1,5	4,9	22,7	34,7	43,6	61,1
	2,2	9,0	18,3	42,6	63,4	62,8
**icatibant**	2,3	5,9	6,4	10,0	28,4	40,9
	6,7	5,8	8,1	9,8	37,0	37,4
	2,8	7,7	4,9	5,4	10,8	23,7
	1,4	1,3	3,0	2,9	6,0	16,3
	4,1	4,3	3,8	4,8	10,2	33,0
	3,0	2,8	3,8	4,5	16,9	29,5
**enalaprilat**	1,9	19,2	16,0	40,9	55,9	66,9
	2,1	5,7	17,8	39,3	47,7	73,8
	0,4	9,8	17,8	35,2	55,7	61,2
	3,3	5,3	11,1	26,0	46,1	60,0
	0,0	3,4	16,0	28,7	35,9	50,2
	3,0	4,8	15,7	29,5	42,4	54,1
**pyrilamine**	4,4	11,0	22,5	40,4	44,2	47,6
	4,0	5,7	21,0	22,6	34,9	40,8
	0,3	6,6	57,0	38,9	34,6	35,5
	0,6	7,4	27,0	40,1	70,7	67,6
	2,4	9,9	14,8	56,2	59,8	59,3
	5,6	15,7				

Fig. 7B

antagonist→<br>agonist↓	control	pyrilamine
BSA vehicle	-1.6<br>7.4<br>1.7<br>2.3<br>1.3<br>1.1<br>0.7<br>1.9	-3.2<br>6.2<br>3.9<br>1.7<br>5.7<br>1.5<br>0.6<br>3.5<br>1.6<br>2.2
histamine	56.9<br>46.2<br>40.0<br>21.6<br>38.4<br>38.7<br>47.2	10.6<br>43.2<br>19.2<br>5.3<br>15.5<br>25.3<br>43.6<br>15.5

Fig. 7C : dose-response curve: ΔMAP, BK

Dose fmol/kg→	0	24	94	378	1510	6040
replicates→	4,2	4,8	5,2	10,2	17,5	28,4
	0,1	4,1	10,9	17,0	57,3	82,3
	4,4	5,9	12,5	21,9	31,0	36,4
	2,8	4,9	4,7	12,3	26,1	32,0
	4,5	3,7	6,1	9,8	26,9	28,8
	2,6	6,2	5,2	7,1	14,5	21,1
	1,6	2,8	5,4	10,6	22,9	42,6
	5,0	3,8	8,1	9,1	25,5	32,1
	3,8	7,3	10,6	14,8	22,1	65,8

Fig. 7D : $t_{1/2}$ recovery

agonist→	BK					MK				
Dose fmol/kg→	24	94	378	1510	6040	11	46	184	734	2770
replicates→	11.1	10.2	12.8	13.8	15.4	9.1	13.23	46.7	83.2	293.8
	7.1	10.2	12	12.9	18.6	10	13.2	16.8	53.5	219.8
	7	8	9.2	11	13.7	5.5	13.4	15.6	31	76.8
	5.9	9.8	10.6	11.4	13.4	9.1	21	24.7	41.3	136.3
	7.8	9.6	11.1	12.3	15.3	11.1	22.9	35.9	52	221
	9.7	7.5	9.5	10.0	10.7	10	12.7	14.9	38.3	107.6
	5.9	10.9	19.1	20.6	30.9	11	24.3	207.2	23.3	186
	5.7	10.0	7.8	15.2	14.9	15.4	39.9	26.5	36.5	114.6
	7.7	11.8	8.9	16.8	18.2					

Fig. 7E : tachycardia

agonist→	BK						MK					
Dose fmol/kg→	0	24	94	378	1510	6040	0	11	46	184	734	2770
replicates→	0	0	4.1	14.9	24.1	43.2	0	4.9	32	44.4	44.2	44.5
	0	11.3	19.9	27.1	26.4	26.1	0	0	10.1	60.5	71.1	62.8
	0	0	9.5	11.6	24.5	65.7	0	7.7	20.9	47.3	46.2	65.5
	0	5.9	26.9	25.3	37.1	83.2	0	0	12	19.8	35.9	36.8
	0	0	22.3	54.3	66.8	104	0	12.9	16.3	31.9	75	60.7
	0	0	9.8	13.4	55.1	92.1	0	0	14	32.1	7.5	48.7
	0	0	0	0	8.3	0	0	19.7	21.1	76.7	120.7	81
	0	0	3.5	20.4	39.2	49.8	0	1.6	20.6	67.9	111.7	68
	0	0	8.1	14.5	37.5	36.9						

Fig. 8 : hypotension

BSA		BK		MK	
control	icatibant	control	icatibant	control	icatibant
0.5	-0.7	34.9	7.5	57.2	57.2
1.0	1.1	32.6	21.4	59.9	59.9
0.5	2.8	44.7	21.9	51.9	51.9
1.1	-1.5	32.8	5.4	45.7	45.7
-1.6	2.7	39.5	13.1	69.6	69.6
1.8	-1.8	32.1	8.8	38.3	38.3
-1.1		43.0		58.6	58.6
0.4		43.1		58.0	58.0

Fig. 8 : vasodilation

BSA		BK		MK	
control	icatibant	control	icatibant	control	icatibant
0.5	1.3	3.8	0.4	7.2	0.8
-0.3	0.0	5.1	1.5	7.6	1.6
-0.7	0.2	4.8	0.0	4.7	0.4
0.3	0.2	6.0	0.4	11.6	1.0
0.1	-0.6	5.6	1.4	9.3	1.6
0.5	0.5	4.9	0.4	4.1	0.1
-0.2		3.9		9.0	
1.1		6.1		10.3	

Fig. 8 : tachycardia

BSA		BK		MK	
control	icatibant	control	icatibant	control	icatibant
0.0	0.0	37.0	2.6	61.0	0.0
1.5	0.0	39.9	5.8	56.3	5.6
1.2	4.0	7.6	0.0	0.6	14.8
3.4	1.3	25.3	8.8	70.1	9.9
0.0	0.0	35.7	19.6	68.2	10.4
0.0	0.0	37.2	9.5	39.9	0.0
0.0		44.1		54.6	
0.0		22.2		34.0	
